# Sustainability and Climate Change Awareness, Attitudes, and Perceptions in Radiology: A Cross‐Sectional Study in Kuwait

**DOI:** 10.1002/hsr2.72187

**Published:** 2026-03-22

**Authors:** Asseel Khalaf, Eman Alawdhi, Mohammad Rawashdeh, Magdi A. Ali, Layla Ghadanfar

**Affiliations:** ^1^ Radiologic Sciences Department, College of Allied Health Sciences Kuwait University Kuwait City Kuwait; ^2^ Medical Imaging Sciences, College of Health Sciences Gulf Medical University Ajman United Arab Emirates; ^3^ Jordan University of Science and Technology Irbid Jordan

**Keywords:** carbon footprint, diagnostic radiography, green imaging, healthcare sustainability, medical imaging

## Abstract

**Introduction:**

Medical imaging contributes to healthcare‐related environmental impacts due to the energy‐ and resource‐intensive nature of diagnostic technologies. Understanding radiology professionals' awareness, attitudes, and perceptions of sustainability is essential to inform education and departmental strategies, particularly in regions where evidence is limited. This study evaluated the awareness, attitudes, and perceptions of radiology professionals in Kuwait regarding sustainability and green imaging.

**Methods:**

A cross‐sectional online survey was conducted among radiology professionals working in Kuwait (*n* = 186). The questionnaire assessed demographics, sustainability‐ and climate‐related training, perceptions of green imaging, and perceived institutional challenges. Open‐ended questions were included to explore perceived barriers and opportunities for sustainable imaging.

**Results:**

Most participants reported high awareness of climate change and its relevance to healthcare; however, formal training in sustainability (30.1%) and recycling (24.7%) was limited. A statistically significant difference by gender was observed in perceptions of climate change impacts (*p* < 0.05). Qualitative findings identified key barriers to sustainable imaging, including a lack of institutional policies, limited leadership support, and insufficient guidance and resources for implementation.

**Conclusion:**

Radiology professionals in Kuwait demonstrate positive awareness and attitudes toward sustainability; however, implementation remains constrained by educational and institutional gaps. Strengthening sustainability‐focused training, leadership engagement, and departmental policy frameworks may facilitate more effective integration of sustainability within radiology services.

## 1 Introduction

Climate change represents significant risks to human health, and healthcare systems themselves contribute to environmental impacts through energy use, resource consumption, and waste generation [1, 2, 3, 4, 5]. Moreover, within healthcare, radiology is among the most resource‐ and energy‐intensive services due to the widespread use of advanced imaging technologies [6, 7]. Diagnostic imaging contributes to environmental impacts across the imaging life cycle, including equipment manufacture, transport, energy‐intensive operation, and waste management [7]. Therefore, its increasing utilization raises growing concerns about its environmental impact.

Modalities, mainly magnetic resonance imaging (MRI) and computed tomography (CT), are particularly energy demanding, while ultrasound has a comparatively lower environmental footprint. A single MRI scan can produce around 17.5 kg of CO₂‐equivalent emissions, while a CT scan produces approximately 9.2 kg [8, 9]. MRI has the highest environmental impact, followed by CT and single‐photon emission computed tomography (SPECT), while ultrasound has the lowest environmental impact. These differences primarily reflect variations in energy consumption. For context, a single MRI scan can produce CO₂ emissions equivalent to driving 145 km in a conventional internal combustion engine vehicle, compared to 76 km for a CT scan and just 4 km for an ultrasound [8]. In addition, imaging modalities also generate substantial heat loads, which might contribute to occupational heat stress. Evidence from occupational health research indicates that prolonged exposure to heat can negatively affect well‐being and productivity and increase the risk of adverse long‐term health outcomes [10].

Within the healthcare sector, radiology ranks among the highest consumers of electricity owing to its extensive use of technologically advanced imaging systems. The energy requirements of various imaging modalities, such as MRI, CT, ultrasound, and workstation computers, have been extensively documented [11, 12, 13]. Moreover, diagnostic imaging contributes to environmental impacts beyond energy use, notably through the release of iodinated contrast agents used for CT imaging and gadolinium‐based agents for MRI into water systems following patient excretion [14, 15]. Additionally, the rapid growth of artificial intelligence (AI) in radiology introduces a dual environmental challenge: training and deploying AI models, along with large‐scale data storage and cloud infrastructure, require substantial computational resources and contribute to greenhouse gas emissions [16, 17]. Emerging literature on “green AI” highlights the importance of energy‐efficient model development, responsible data center selection, and transparent reporting of AI‐related emissions. At the same time, AI has the potential to enhance sustainability by improving scanner efficiency, reducing low‐value imaging, optimizing scheduling, and minimizing contrast use. These evolving considerations underscore the need for sustainability awareness across all dimensions of modern radiology practice [17, 18].

The rising demand for advanced imaging technologies highlights the need for sustainable imaging practices. Yet, sustainability is rarely included in formal quality metrics, limiting opportunities for systematic improvement. “Green imaging” refers to environmentally responsible approaches within radiology, like the use of energy‐efficient equipment, optimized imaging protocols, and effective waste management [1, 9, 17]. Despite international efforts to promote sustainability in healthcare, there is limited awareness among healthcare professionals about green imaging and sustainability. Additionally, inefficient practices and the absence of institutional policies are considered significant barriers to implementation.

While research on environmental sustainability in radiology is still emerging globally, a growing body of evidence has begun to document sustainability awareness, attitudes, and perceived barriers among radiology professionals in the Gulf region. Recent studies from the Gulf region have begun to examine sustainability awareness, attitudes, and perceived barriers among radiology professionals, highlighting important challenges related to training, leadership, and institutional support. Studies from Saudi Arabia and the United Arab Emirates have reported generally positive attitudes toward sustainability alongside persistent gaps in formal training, institutional leadership, and policy support, highlighting challenges in translating awareness into routine practice [19, 20]. Multi‐country studies that include Gulf Countries have demonstrated regional variability in sustainability engagement among radiographers, with institutional resources and educational initiatives playing a critical role in shaping implementation [21]. However, published evidence specifically examining radiology professionals in Kuwait remains limited. A lack of research on these issues in Kuwait underscores an evident gap in the existing literature.

This study aimed to evaluate the awareness, attitudes, and perceptions of radiology professionals in Kuwait towards sustainability and green imaging practices.

## 2 Materials and Methods

### 2.1 Study Design

This study employed a questionnaire‐based survey adapted from a previously developed and validated instrument, which has been used in previously published studies examining sustainability‐related awareness, perceptions, and barriers among radiology professionals [22, 23]_._ The survey targeted radiology professionals working in government hospitals in Kuwait.

A Microsoft Forms (Microsoft Inc., Redmond, WA) was used to create an online questionnaire. The survey consisted of 36 closed‐ended questions presented in multiple formats, including multiple‐choice questions with text‐entry options and drop‐down selections. The survey is structured into five key sections: demographic information, training related to global warming and climate change, views on sustainability practices, challenges to sustainability, and radiography‐related practices. These sections collectively capture respondents' awareness, attitudes, and perceptions toward sustainability, with the latter two sections providing insight into perceived barriers and self‐reported experiences. Responses in the main sections were measured using a 5‐point Likert scale ranging from strongly disagree to strongly agree. In items referring to “recycling” of radiographic, nuclear medicine, or contrast‐related waste, the term was used to reflect participants' perceptions of environmentally responsible waste management practices within imaging departments. Additionally, the survey included two open‐ended questions to collect qualitative insights from participants. The questionnaire was distributed through a multimodal approach, including in‐person hospital visits where participants provided Quick Response (QR) codes, as well as selected online channels. The questionnaire was administered on a secure online platform, and the research team had exclusive access to the securely stored, anonymized data throughout the entire study. The inclusion criteria were male and female adults, including radiographers and radiologists aged 18 years or older, working in the governmental radiology department in Kuwait's hospitals.

### 2.2 Ethical Approval

Ethical approval was obtained from the Health Sciences Centre (HSC) Ethical Committee and the Kuwait Ministry of Health (FAHS/99). All the participants provided informed consent for participation before starting the survey. Participation in the study was voluntary. The data collection was between January 2025 and March 2025.

### 2.3 Data Analysis

The responses were exported to a Microsoft Excel and subsequently analyzed using the Statistical Package for Social Sciences (SPSS Inc., IBM, New York, USA). Categorical data are presented as numbers and percentages. The Chi‐square test was used to examine associations between categorical variables. Alternatively, the Fisher Exact correction test was used when more than 20% of the cells had an expected count of less than five, with *p* values < 0.05 demonstrating statistical significance. Subgroup comparisons were conducted across predefined demographic variables (age, years of experience, gender, and nationality) to explore potential associations with sustainability perceptions. Continuous variables are presented as mean ± standard deviation (SD).

Open‐ended responses were analysed using an inductive thematic analysis approach. The analysis followed a structured process including familiarization with the data, initial coding of meaningful segments, identification of recurring patterns, and development of broader themes. Coding was conducted manually by the lead researcher, with iterative review of responses to refine and consolidate emerging themes.

## 3 Results

### 3.1 Demographics

**Figure 1:**
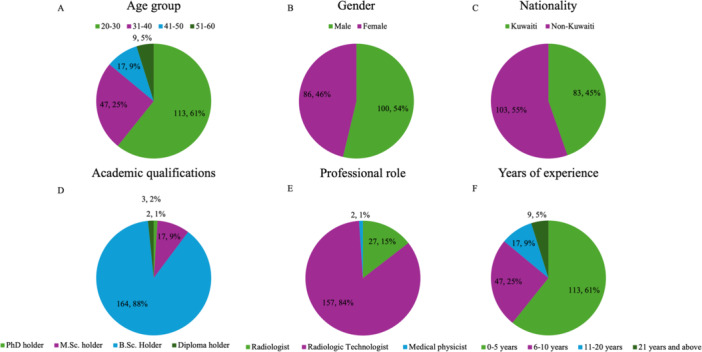
Distribution of demographic characteristics among study participants (*n* = 186). (A) age groups, (B) gender, (C) nationality distribution (Kuwaiti vs. non‐Kuwaiti), (D) academic qualification levels, (E) distribution by professional role, (F) years of experience. Values are shown as numbers and percentages.

A total of 186 participants completed the questionnaire, with ages ranging from 20 to 60 years. Of these, 53.8% were male, and 46.2% were female (Figure 1). Regarding nationality, 55.4% of participants were non‐Kuwaiti, and 44.6% were Kuwaiti. The participants' academic qualifications were as follows: 88.2% held a Bachelor of Science (BSc) degree, 9.1% had a Master of Science (MSc) degree, 1.6% held a Diploma, and 1.1% held a Doctor of Philosophy (PhD) degree (Figure 1). Regarding the professional roles, 84.4% of participants were radiologic technologists, 14.5% were radiologists, and 1.1% were medical physicists. Most of the participants (52.7%) had 0–5 years of experience, followed by 21.5% with 6–10 years, 17.2% with 11–20 years, and 8.6% with more than 21 years of experience (Figure 1).

### 3.2 Sustainability Training

**Figure 2:**
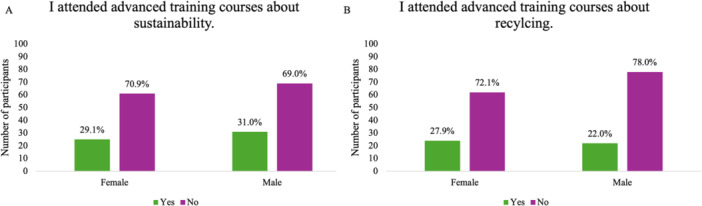
Percentage of participants who attended training courses, categorized by gender. (A) Attendance in advanced sustainability courses, and (B) attendance in recycling‐related courses.

Only 30.1% of the participants had attended advanced courses on sustainability, and an even smaller proportion had participated in recycling courses (24.7%), with no significant gender differences observed (Figure 2). When participants were asked to identify their sources of information regarding sustainability and recycling, almost half of the participants reported having no prior information (49.5%). Among those who cited a source, training courses were the most frequently mentioned, followed by conferences and the internet.

### 3.3 Perceptions of Global Warming and Local Climate Change

Most of the participants (84.4%) expressed confidence in their knowledge of climate change and its impact on the environment. Moreover, most participants (90.4%) believed that climate change had already occurred, and 74.7% believed that it had already happened in their local region (in Kuwait) (Supporting Information S1: Table 1). Additionally, 85% of participants expressed concern about the negative consequences of global climate change. Most participants expressed strong concerns about the impacts of climate change across multiple domains (Supporting Information S1: Table 1). Specifically, 80.6% strongly agreed that climate change would lead to more frequent extreme weather events, including droughts, storms, floods, and hurricanes. A large proportion (91.4%) believed it would harm human health, and 86% anticipated consequences for agricultural production. The emergence and re‐emergence of infectious diseases due to climate change were acknowledged by 85.4% of participants. Furthermore, statistically significant gender differences (*p *< 0.05) were identified when they were asked about the levels of agreement concerning the influence of climate change on human health, agricultural productivity, sea‐level rise, and the potential emergence and re‐emergence of infectious diseases (Supporting Information S1: Table 1).

### 3.4 Sustainability Practices in Radiology

Most respondents (68.3%) believed that sustainability practices within their departments require improvement (Table 1). In response to concerns about climate change, 61.8% of the participants reported making changes to their personal behavior (Table 1). Furthermore, 65.1% agreed that staff have a responsibility to consider the environmental impact of imaging services.

**Table 1:** Relationship between gender and sustainability practice in radiology, *n* = 186.

Response	Total *n* (%)	Female *n* (%)	Male *n* (%)	*p* value	Effect size (Cramer's *V*)
Department could do more to improve its sustainability					
Strongly agree	31 (16.7)	16 (8.6)	15 (8.1)	0.669	0.119
Agree	96 (51.6)	41 (22.0)	55 (29.6)		
Neutral	50 (26.9)	24 (13.0)	26 (14.0)		
Disagree	8 (4.3)	5 (2.7)	3 (1.6)		
Strongly disagree	1 (0.5)	0 (0.0)	1 (0.5)		
Personal behavior changes due to climate concern					
Strongly agree	29 (15.6)	11 (5.9)	18 (9.7)	0.313	0.160
Agree	86 (46.2)	41 (22.0)	45 (24.2)		
Neutral	56 (30.1)	28 (15.1)	28 (15.1)		
Disagree	13 (7.0)	4 (2.2)	9 (4.8)		
Strongly disagree	2 (1.1)	2 (1.1)	0 (0.0)		
Responsibility for awareness of environmental impact					
Strongly agree	31 (16.7)	14 (7.5)	17 (9.1)	0.761	0.109
Agree	90 (48.4)	39 (21.0)	51 (27.4)		
Neutral	59 (31.7)	31 (16.7)	28 (15.1)		
Disagree	5 (2.7)	2 (1.1)	3 (1.6)		
Strongly disagree	1 (0.5)	0 (0.0)	1 (0.5)		
Recycling at home					
Strongly agree	24 (13.0)	13 (7.0)	11 (5.9)	0.226	0.171
Agree	77 (41.4)	29 (15.6)	48 (25.8)		
Neutral	58 (31.2)	32 (17.2)	26 (14.0)		
Disagree	24 (13.0)	10 (5.4)	14 (7.5)		
Strongly disagree	3 (1.6)	2 (1.1)	1 (0.5)		
Recycling of imaging and contrast‐related waste in hospital					
Strongly agree	23 (12.4)	12 (6.5)	11 (5.9)	0.133	0.195
Agree	39 (21.0)	11 (5.9)	28 (15.1)		
Neutral	71 (38.2)	36 (19.4)	35 (18.8)		
Disagree	43 (23.1)	23 (12.4)	20 (10.8)		
Strongly disagree	10 (5.4)	4 (2.2)	6 (3.2)		
Willingness to recycle radiographic and nuclear medicine waste					
Strongly agree	34 (18.3)	13 (7.0)	21 (11.3)	0.575	0.126
Agree	70 (37.6)	34 (18.3)	36 (19.4)		
Neutral	44 (23.7)	24 (13.0)	20 (10.8)		
Disagree	30 (16.1)	12 (6.5)	18 (9.7)		
Strongly disagree	8 (4.3)	3 (1.6)	5 (2.7)		

*Note: p* values were obtained using *χ* ^2^ or Fisher's exact tests. Effect size reported as Cramer's *V*.

In terms of recycling, respondents reported higher engagement in personal recycling behaviors compared with perceived recycling practices within hospital settings. While just over half of the participants (54.4%) reported actively recycling at home, views on hospital‐based recycling practices were more divided: with fewer (33.4%) respondents agreeing that imaging and contrast media waste is typically recycled. Notably, 55.9% of respondents expressed support for expanding recycling efforts to include radiographic waste, with no statistically significant gender differences observed (Table 1).

### 3.5 Barriers to Sustainable Practices in Radiology

**Figure 3:**
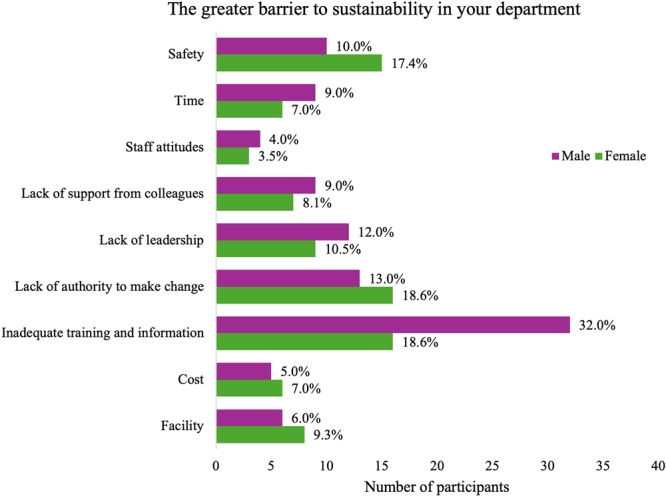
Participants' perceptions of the most significant barriers to sustainability in the radiology department, categorized by gender.

Among the barriers identified, inadequate training and lack of information emerged as the most frequently reported concern (25.8%, Figure 3). Other significant obstacles reported included limited authority to implement changes (15.6%) and a lack of leadership support (11.3%). Safety concerns (13.4%) and infrastructural or facility limitations (7.5%) were also highlighted as contributing to the complexity of implementing sustainability initiatives. Additional challenges included time constraints (8.1%), financial considerations (6%), and staff attitudes (3.8%) (Figure 3).

Broad barrier categories were used to capture respondents' perceived constraints across diverse clinical settings. In this context, time reflects workload and workflow pressures, safety concerns related to radiation protection, infection control, or waste handling, and facility refers to the availability of infrastructure and institutional systems supporting sustainable practices.

### 3.6 Thematic Analysis: Promoting Sustainability and Green Imaging in Radiology

Thematic analysis of the open‐ended responses from 186 participants identified four primary themes with several subthemes. These findings provide richer insight into the participants' views on sustainability and green imaging in Kuwait.

Theme 1: Awareness and education

Participants widely emphasized the need to enhance awareness and formal education on sustainability. Subthemes: Lectures and training programmes. Most of the participants highlighted the necessity of frequent workshops, awareness programmes, and ongoing education in sustainability. “Do awareness lectures to all staff.” “By giving more lectures.” Knowledge gaps: Some expressed uncertainty or lack of training, suggesting an urgent need for structured educational initiatives. “I honestly don't know; we're not trained well in this topic.”

Theme 2: Institutional policies and leadership

Respondents emphasized the importance of institutional support in leading sustainable practice. Subthemes: Supportive leadership: Several responses called for stronger leadership commitment to sustainability goals. “Leadership should prioritize and model sustainable practices.” “Lead by example.” Hospital‐wide policy enforcement: Participants recommended formal policies, recycling programs, and designated sustainability committees. “More regulation on it; leadership must take care of it.” “Hospital policy required.”

Theme 3: Practical environmental actions

Participants described actionable measures that could be immediately implemented within radiology departments. Subthemes: Recycling and waste reduction: Suggestions frequently focused on minimizing paper usage and promoting digitization. “Recycle, digitize paper reports and pictures.” “Reduce waste, recycle materials.” Energy efficiency and equipment use: Participants emphasized eco‐friendly behavior and the adoption of energy‐efficient imaging devices. “Use energy‐efficient machines, do not scan unnecessarily.”

Theme 4: Cultural and behavioral change

Responses also reflected the need for broader cultural transformation within healthcare settings. Subthemes Teamwork and involvement: Collaboration across teams was viewed as essential to sustaining green practices. While participants frequently emphasized teamwork and collaboration as essential for sustaining green practices, many responses remained general or non‐specific, like calls to “Listen to each other, work as a team, respect each other.” Rather than outlining specific or actionable strategies, these statements may indicate uncertainty or insufficient guidance regarding the translation of sustainability into routine clinical practice. The broadly framed nature of these responses might indicate uncertainty regarding how sustainability initiatives can be operationalized in daily clinical practice. This lack of specificity may also reflect a perceived limitation in individual agency, suggesting that staff may be willing to support sustainability efforts but require clearer institutional direction, leadership engagement, and structured implementation pathways to translate intentions into practice. In addition, some respondents highlighted the importance of embedding sustainability within everyday workplace values and environments, for example, through suggestions to build a green culture: “Provide some green leaf areas in the hospital for a fresh atmosphere.”

## 4 Discussion

With the expanding use of medical imaging procedures, the healthcare sector faces a growing challenge: the environmental impact of diagnostic imaging. Although this issue has been highlighted in the literature [8, 12], evidence from the region remains limited. This study provides the first nationwide assessment of radiographers' perspectives on sustainability and green imaging practices in Kuwait, offering a baseline for future policy and educational planning. The findings highlight clear gaps in knowledge, confidence, and institutional support, indicating the need for more structured efforts moving forward.

The results indicated that most participants have little formal training in sustainability and recycling, with a significant lack of prior information on these topics. This highlights the need for more accessible education and awareness programs to strengthen knowledge and practices in this area. In the present study, although most participants expressed confidence in their understanding of climate change and its effects on human health and the environment, only a minority reported having formal training in sustainability. This limited exposure aligns with findings by Rawashdeh et al [22], who identified inadequate training and information as persistent barriers to the adoption of green imaging practices. Additionally, the findings revealed that most participants recognize that climate change is already affecting their local region (in Kuwait). Moreover, there is widespread concern regarding the negative consequences of climate change across multiple domains, including extreme weather events, human health, agricultural productivity, and the potential emergence or re‐emergence of infectious diseases. Notably, differences in perceptions were observed across demographic groups, with gender being the only variable to demonstrate statistically significant differences in the present analysis. Although gender‐related differences reached statistical significance, their practical importance likely reflects variations in access to training opportunities, professional responsibilities, or communication pathways rather than intrinsic differences between individuals. Accordingly, sustainability education and departmental initiatives should prioritise equitable access to training, leadership engagement, and institutional support to ensure that sustainability awareness and implementation efforts reach all staff groups.

Although gender was the only demographic variable demonstrating statistically significant differences, age and years of professional experience remain important contextual factors when interpreting sustainability engagement. The lower participation observed among older and more experienced professionals may have reduced the statistical power to detect potential age‐related differences. It may also indicate an engagement gap that warrants targeted educational and institutional initiatives. This pattern may reflect differences in engagement with sustainability initiatives, access to formal training opportunities, or perceived relevance within established clinical practice settings. These findings align with emerging evidence that institutional hierarchy and access to professional development strongly shape sustainability engagement in clinical environments [21]. Future studies using larger and more balanced samples should further explore whether professional seniority influences sustainability awareness, training access, or implementation behaviors.

The findings indicate that participants are acknowledging a personal and professional responsibility to address environmental impacts. Opinions were mixed regarding hospital‐based recycling, particularly for waste from imaging and contrast media. Nevertheless, there is clear support for expanding recycling initiatives within radiology departments, suggesting an opportunity to strengthen sustainability efforts and promote environmentally responsible practices among staff. These findings are consistent with global efforts promoting eco‐friendly imaging practices, including the use of energy‐efficient equipment, optimization of imaging protocols, and responsible waste management [1, 24].

The results highlighted several challenges to implementing sustainability initiatives in the field of radiology. The most prominent barriers were insufficient training and a lack of information, followed by limited authority to enact changes and inadequate leadership support. These obstacles correspond with prior reports emphasizing the importance of organizational culture, leadership engagement, and resource allocation in advancing sustainability within healthcare settings [9, 25]. Another concerning observation was the low prevalence of formal recycling systems for imaging and contrast media waste. Given the potentially hazardous nature of certain imaging wastes, proper disposal and recycling are critical for environmental protection and public health [24].

These findings align with recent multi‐society guidance, including the joint call to action published in Radiology, which emphasizes integrating sustainability into routine radiology practice through leadership‐driven strategies, structured education, energy‐conscious workflow planning, and waste‐reduction initiatives [26]. Such recommendations reinforce the importance of formal training, designated sustainability leadership, and policy‐level support, areas identified as gaps in the present study. Furthermore, several countries have adopted life‐cycle–informed approaches to procurement and workflow planning, which have been shown to reduce waste and energy use at the departmental level [26, 27, 28, 29].

Moreover, the primary challenge the medical imaging sector faces is maintaining high diagnostic standards while simultaneously reducing the carbon footprint of its practices. This requires a proactive approach that engages both industry partners and patients in advancing sustainable healthcare [30]. A survey conducted in Italy found that 93% of physicians considered the CO_2_ emissions associated with each procedure to be an important consideration when making decisions regarding cardiac imaging [31]. The industry here plays a critical role in developing advanced medical imaging technologies that aim to reduce carbon emissions while maintaining diagnostic resolution. The literature suggests that informed consent documents should present radiologic exposure in terms of chest x‐ray equivalents, alongside the carbon footprint expressed in kg of CO₂ emissions. To enhance patient understanding, these values can be compared with everyday activities, such as the distance travelled by car. Presenting such comparisons offers patients and clinicians a clearer understanding of the environmental burden associated with imaging procedures, which is often underestimated in clinical practice [12].

Our qualitative findings support explaining the survey results and offer a clearer picture of what is driving current attitudes toward sustainability in radiology. Many respondents expressed a willingness to adopt greener practices but reported lacking adequate training, practical guidance, and institutional support. In addition, several responses were broadly framed, suggesting uncertainty regarding how sustainability goals can be translated into routine professional practice rather than a lack of interest or motivation. Others highlighted the need for hospital‐wide strategies, clearer policies, and a stronger leadership role to move sustainability from an individual effort to an organized departmental priority. Likewise, a European survey showed that while radiology professionals recognize the importance of environmental stewardship, they often lack guidance, leadership, and departmental policy to implement sustainable procedures [28]. These observations are consistent with our qualitative findings, which underline the essential role of leadership, structured training, and coordinated organizational action in driving meaningful improvements.

These insights make it evident that the issue is not a lack of awareness alone, but rather the absence of structured systems that enable change. Taken together, the qualitative data show that radiology professionals in Kuwait are open to sustainable practices, but meaningful progress will require leadership commitment, educational initiatives, and coordinated departmental action. These findings align with emerging evidence from the Gulf region, which indicates that radiology professionals generally demonstrate positive attitudes toward sustainability but experience limited access to formal training and ongoing institutional barriers to implementation. Collectively, regional studies suggest that the principal challenge lies not in awareness itself, but in translating willingness into routine clinical practice through effective governance structures, adequate resources, and standardized policies [19, 20, 21].

Based on current evidence and the barriers reported by participants in this study, several practical actions can be implemented at the departmental level. First, radiology departments can establish a “Green Radiology” committee with defined responsibilities (e.g., monitoring energy use, coordinating waste initiatives, and staff communication) and integrate sustainability targets into departmental quality and safety meetings [2]. Second, departments can adopt energy‐efficiency protocols, including powering down workstations and non‐essential equipment when not in use, optimizing scanner standby settings, and scheduling workflows to reduce unnecessary time; where clinically appropriate, lower‐energy modalities should be prioritized and repeat imaging minimized through protocol optimization and quality assurance. Third, departments can implement waste‐stream segregation and recycling pathways tailored to radiology and transition from disposable to reusable supplies where infection control allows [32]. Fourth, radiology services in Kuwait can embed sustainability into training and continuing professional development. Finally, sustainability should be incorporated as a formal quality measure, and manufacturers are encouraged to provide Environmental Product Declarations (EPDs) derived from life cycle assessments (LCAs) for imaging equipment [33].

Moreover, integrating sustainability principles into curricula, developing national guidelines and standardized protocols, and establishing green leadership and continuous professional development programs for radiology professionals are recommended.

Several limitations must be acknowledged. As an exploratory cross‐sectional survey using voluntary participation, the sample may not fully represent all radiology professionals in Kuwait. A formal a priori sample size calculation was not performed, and subgroup analyses were exploratory in nature; therefore, findings should be interpreted as baseline observations rather than confirmatory evidence. Although a previously published survey instrument was used, the reliance on self‐reported data may introduce response and social desirability bias, as participants' reported awareness and attitudes may not fully reflect actual clinical practices. In addition, participant demographics were imbalanced, with radiographers comprising most respondents while radiologists were underrepresented, which may limit role‐specific inference. The lower participation rate among older and more experienced professionals may have reduced the ability to detect age‐ or experience‐related differences. Formal psychometric validation of the questionnaire was not conducted within the Kuwaiti context. Given the exploratory aim of the study, analyses were performed at the item level. Future research may undertake further validation and include larger, more balanced samples. Finally, the considerable length of the survey may have contributed to respondent fatigue, potentially affecting response quality.

In summary, although radiologic professionals in Kuwait demonstrate growing awareness of environmental sustainability, this awareness has not yet fully translated into consistent practices. Future efforts in Kuwait should prioritise integrating sustainability concepts into the education and training curricula for radiology professionals. Future efforts should focus on integrating sustainability concepts into the curricula for radiology professionals, establishing national green imaging standards, and empowering imaging professionals through enhanced authority and institutional support. Moreover, fostering interdepartmental collaboration and sustained leadership engagement will be crucial to addressing existing barriers and promoting environmentally sustainable practices in the field of radiology.

## 5 Conclusion

The findings of this study reflect growing international concern about the environmental footprint of medical imaging and underscore the importance of strengthening sustainability practices as radiology's clinical role continues to expand. Integrating environmental responsibility into imaging practices is increasingly important, and aligning local initiatives in Kuwait with international frameworks will be vital to supporting broader climate action goals.

Addressing potential differences in engagement across career stages through tailored sustainability training, mentorship programmes, and leadership‐supported initiatives may enhance participation and facilitate more effective integration of sustainable practices within radiology departments. By promoting leadership engagement, advancing education, and adopting evidence‐based green imaging strategies, radiology departments can meaningfully reduce the environmental footprint of healthcare and contribute to a more resilient and sustainable healthcare system.

## Author Contributions

**Asseel Khalaf:** conceptualisation, methodology, formal analysis, investigation, resources, data curation, writing – original draft, writing – review and editing. **Eman Alawdhi:** formal analysis, investigation, resources, data curation, and writing –original draft. **Mohammad Rawashdeh:** conceptualisation, methodology, validation, writing – review and editing. **Magdi A. Ali:** conceptualisation, methodology, validation, writing – review and editing. **Layla Ghadanfar:** project administration, writing – review and editing, visualisation, and supervision.

## Funding

The authors received no specific funding for this work.

## Disclosure

All authors have read and approved the final version of the manuscript. Dr. Khalaf had full access to all the data in this study and takes complete responsibility for the integrity and accuracy of the data and the analysis.

## Conflicts of Interest

The authors declare no conflicts of interest.

## Transparency Statement

The corresponding author, Asseel Khalaf, affirms that this manuscript is an honest, accurate, and transparent account of the study being reported; that no important aspects of the study have been omitted; and that any discrepancies from the study as planned (and, if relevant, registered) have been explained.

## Supporting information

Supplementary Table updated 1.

## Data Availability

The data that support the findings of this study are available from the corresponding author upon reasonable request.
