# Supplementary material for: Sustainability and Climate Change Awareness, Attitudes, and Perceptions in Radiology: A Cross‐Sectional Study in Kuwait
Source: Health Sci Rep. 2026 Mar 22;9(3):e72187. doi: 10.1002/hsr2.72187 (PMC13098053; doi:10.1002/hsr2.72187)
Supplement: Supplementary file 1 — Supplementary Table updated 1. [file HSR2-9-e72187-s001.docx]

**Supplementary Table 1:** Relationship between gender and perception of climate change and global warming, n=186.

|  | **Total (n=186)** | | **Female (n=86)** | | **Male (n=100)** | | **p-value** | **Effect size (Cramer’s V)** |
| --- | --- | --- | --- | --- | --- | --- | --- | --- |
|  | N | % | N | % | N | % |  |  |
| **I am well-informed about climate change and the impact that human action has on the natural world** | | | | | | |  |  |
| Strongly Agree | 68 | 36.6% | 27 | 14.5% | 41 | 22% | 0.421 | 0.125 |
| Agree | 89 | 47.8% | 43 | 23.1% | 46 | 24.7% |  |  |
| Neutral | 26 | 14% | 15 | 8.1% | 11 | 5.9% |  |  |
| Disagree | 0 | 0% | 0 | 0% | 0 | 0% |  |  |
| Strongly Disagree | 3 | 1.6% | 1 | 0.5% | 2 | 1.1% |  |  |
| **I am concerned about the climate change, and the impact people are having on the environment** | | | | | | |  |  |
| Strongly Agree | 61 | 32.8% | 28 | 15.1% | 33 | 17.7% | 0.272 | 0.166 |
| Agree | 97 | 52.2% | 42 | 22.6% | 55 | 20.6% |  |  |
| Neutral | 25 | 13.4% | 15 | 8.1% | 10 | 5.4% |  |  |
| Disagree | 1 | 0.5% | 1 | 0.5% | 0 | 0% |  |  |
| Strongly Disagree | 2 | 1.1% | 0 | 0% | 2 | 1.1% |  |  |
| **Human activities (compared to natural factors) are the main cause of climate change** | | | | | | |  |  |
| Strongly Agree | 64 | 34.4% | 32 | 17.2% | 3 | 17.2% | 0.914 | 0.055 |
| Agree | 93 | 50% | 40 | 21.5% | 53 | 28.5% |  |  |
| Neutral | 26 | 14% | 13 | 6.9% | 13 | 6.9% |  |  |
| Disagree | 3 | 1.6% | 1 | 0.5% | 2 | 1.1% |  |  |
| Strongly Disagree | 0 | 0% | 0 | 0% | 0 | 0% |  |  |
| **Climate change has already occurred** | | | | | | |  |  |
| Strongly Agree | 63 | 33.9% | 22 | 10% | 54 | 25% | 0.332 | 0.147 |
| Agree | 105 | 56.5% | 49 | 23% | 73 | 34% |  |  |
| Neutral | 15 | 8.1% | 4 | 2% | 11 | 5% |  |  |
| Disagree | 1 | 0.5% | 0 | 0% | 2 | 1% |  |  |
| Strongly Disagree | 2 | 1.1% | 1 | 0% | 1 | 0% |  |  |
| **Climate change can be avoided** | | | | | | |  |  |
| Strongly Agree | 21 | 11.3% | 9 | 4.8% | 12 | 6.5% | 0.542 | 0.129 |
| Agree | 87 | 46.8% | 39 | 21.% | 48 | 25.8% |  |  |
| Neutral | 53 | 28.5% | 25 | 13.4% | 28 | 15.1% |  |  |
| Disagree | 20 | 10.8% | 12 | 6.5% | 8 | 4.3% |  |  |
| Strongly Disagree | 5 | 2.7% | 1 | 0.5% | 4 | 2.2% |  |  |
| **If someone called for it, I would like to join the actual efforts to mitigate climate change** | | | | | | |  |  |
| Strongly Agree | 28 | 15.1% | 15 | 8.1% | 13 | 6.9% | 0.710 | 0.110 |
| Agree | 91 | 48.9% | 42 | 22.6% | 49 | 26.3% |  |  |
| Neutral | 56 | 30.1% | 26 | 13.9% | 30 | 16.1% |  |  |
| Disagree | 8 | 4.3% | 2 | 1.1% | 6 | 3.2% |  |  |
| Strongly Disagree | 3 | 1.6% | 1 | 0.5% | 2 | 1.1% |  |  |
| **I am willing to sacrifice some individual benefit to solve existing problems** | | | | | | |  |  |
| Strongly Agree | 28 | 15.1% | 12 | 6.5% | 16 | 8.6% | 0.446 | 0.136 |
| Agree | 88 | 47.3% | 36 | 19.4% | 52 | 28% |  |  |
| Neutral | 53 | 28.5% | 30 | 16.1% | 23 | 12.4% |  |  |
| Disagree | 15 | 8.1% | 7 | 3.8% | 8 | 4.0% |  |  |
| Strongly Disagree | 2 | 1.1% | 1 | 0.5% | 1 | 0.5% |  |  |
| **I do participate in some environmental protection activities related to climate change** | | | | | | |  |  |
| Strongly Agree | 26 | 14% | 10 | 5.4% | 16 | 8.6% | 0.419 | 0.145 |
| Agree | 73 | 39.2% | 30 | 16.1% | 43 | 23.1% |  |  |
| Neutral | 57 | 30.6% | 28 | 15.1% | 29 | 15.6% |  |  |
| Disagree | 25 | 13.4% | 15 | 8.1% | 10 | 5.4% |  |  |
| Strongly Disagree | 5 | 2.7% | 3 | 1.6% | 2 | 1.1% |  |  |
| **Climate change has happened in my local region** | | | | | | |  |  |
| Strongly Agree | 35 | 18.8% | 17 | 9.1% | 18 | 9.7% | 0.716 | 0.113 |
| Agree | 104 | 55.9% | 45 | 24.2% | 59 | 31.7% |  |  |
| Neutral | 36 | 19.4% | 17 | 9.1% | 19 | 10.2% |  |  |
| Disagree | 10 | 5.4% | 6 | 3.2% | 4 | 2.2% |  |  |
| Strongly Disagree | 1 | 0.5% | 1 | 0.5% | 0 | 0% |  |  |
| **Influences of climate change:** | | | | | | |  |  |
| Increasing extreme weather (storms, floods, droughts, hurricanes, etc.) | | | | | | |  |  |
| Strongly Agree | 48 | 25.8% | 20 | 10.8% | 28 | 15.1% | 0.781 | 0.106 |
| Agree | 102 | 54.8% | 48 | 25.8% | 54 | 29.0% |  |  |
| Neutral | 32 | 17.2% | 15 | 8.1% | 17 | 9.1% |  |  |
| Disagree | 3 | 1.6% | 2 | 1.1% | 1 | 0.5% |  |  |
| Strongly Disagree | 1 | 0.5% | 1 | 0.5% | 0 | 0% |  |  |
| **Affecting human health** | | | | | | |  |  |
| Strongly Agree | 68 | 36.6% | 25 | 13.4% | 43 | 23.1% | 0.048* | 0.180 |
| Agree | 102 | 54.8% | 50 | 26.9% | 52 | 28% |  |  |
| Neutral | 16 | 8.6% | 11 | 5.9% | 5 | 2.7% |  |  |
| Disagree | 0 | 0% | 0 | 0% | 0 | 0% |  |  |
| Strongly Disagree | 0 | 0% | 0 | 0% | 0 | 0% |  |  |
| **Affecting agricultural production** | | | | | | |  |  |
| Strongly Agree | 64 | 34.4% | 25 | 29.1% | 39 | 39% | 0.029* | 0.223 |
| Agree | 96 | 51.6% | 43 | 50% | 53 | 53% |  |  |
| Neutral | 24 | 12.9% | 17 | 19.8% | 7 | 7% |  |  |
| Disagree | 1 | 0.5% | 0 | 0% | 1 | 1% |  |  |
| Strongly Disagree | 1 | 0.5% | 1 | 1.2% | 0 | 0% |  |  |
| **Initiating a natural ecological crisis** | | | | | | |  |  |
| Strongly Agree | 44 | 23.7% | 16 | 8.6% | 28 | 15.1% | 0.163 | 0.155 |
| Agree | 107 | 57.5% | 49 | 26.3% | 58 | 31.2% |  |  |
| Neutral | 33 | 17.7% | 20 | 10.8% | 13 | 7% |  |  |
| Disagree | 2 | 1.1% | 1 | 0.5% | 1 | 0.5% |  |  |
| Strongly Disagree | 0 | 0% | 0 | 0% | 0 | 0% |  |  |
| **Sea-level rise submerging low-lying areas** | | | | | | |  |  |
| Strongly Agree | 40 | 21.5% | 13 | 15.1% | 27 | 27% | 0.036* | 0.212 |
| Agree | 102 | 54.8% | 48 | 55.8% | 54 | 54% |  |  |
| Neutral | 40 | 21.5% | 21 | 24.4% | 19 | 19% |  |  |
| Disagree | 4 | 2.2% | 4 | 4.7% | 0 | 0% |  |  |
| Strongly Disagree | 0 | 0% | 0 | 0% | 0 | 0% |  |  |
| **The increasing threat of infectious disease** | | | | | | |  |  |
| Strongly Agree | 60 | 32.2% | 22 | 11.8% | 38 | 20.4% | 0.022* | 0.215 |
| Agree | 99 | 53.2% | 46 | 24.7% | 53 | 28.5% |  |  |
| Neutral | 26 | 14% | 18 | 9.7% | 8 | 4.3% |  |  |
| Disagree | 1 | 0.5% | 0 | 0% | 1 | 0.5% |  |  |
| Strongly Disagree | 0 | 0% | 0 | 0% | 0 | 0% |  |  |

p-values were obtained using Chi-square or Fisher’s exact tests. Effect size reported as Cramer’s V. *Statistically significant at p<0.05.
